# Sensory Characterization of Iberian Dry-Cured Loins by Using Check-All-That-Apply (CATA) Analysis and Multiple-Intake Temporal Dominance of Sensations (TDS)

**DOI:** 10.3390/foods10091983

**Published:** 2021-08-25

**Authors:** Alberto González-Mohino, Sonia Ventanas, Mario Estévez, Lary Souza Olegario

**Affiliations:** 1 Meat and Meat Products Research Institute (IProCar), Food Technology, University of Extremadura, 10003 Cáceres, Spain; albertogj@unex.es (A.G.-M.); sanvenca@unex.es (S.V.); 2 Department of Food Engineering, Technology Centre, Federal University of Paraiba, Joao Pessoa 58051-900, Brazil; laryolegario@hotmail.com

**Keywords:** Iberian dry-cured loin, Check-all-that-apply (CATA), ideal product, multiple intake Temporal Dominance of Sensations (TDS), drivers of liking

## Abstract

The aim of the present work was to sensorially characterize different commercial categories of Iberian dry-cured loins (varying genetic and feeding background) using a novel dynamic sensory technique, and to explore consumers preferences applying a rapid method. The samples (green label—GL, Cebo de Campo Ibérico; red label—RL, Bellota 50% Ibérico; and black label—BL, Bellota 100%) were analyzed by (i) Check-all-that-apply (CATA) with the evaluation of an ‘Ideal’ dry-cured loin and the overall liking, and by (ii) multiple-intake Temporal Dominance of Sensations (TDS). The CATA results indicated that the sensory characteristics of RL samples were closer to those of the ‘Ideal’ loin. Furthermore, juiciness, marbling, cured flavor, chewiness, persistence, and brightness were selected as ‘must-have’ attributes. Juiciness cured flavor and red color were considered as drivers of liking. TDS results showed that flavor attributes presented the highest dominance rates, with saltiness being the most dominant attribute along BL sample evaluation, and with cured and paprika flavor for GL and RL samples. These sensory technique results displayed the ability to sensorily characterize dry-cured loins, providing different, complementary, and valuable information.

## 1. Introduction

Traditional dry-cured meat products produced from Iberian pigs are highly considered and appreciated by Spanish consumers [1]. Dry-cured loin is considered one of the most valuable pieces obtained from Iberian pigs, due to its sensory properties which are a consequence of both the characteristics of the raw material and the particular ripening conditions [2]. According to Spanish regulation [3], different commercial categories of dry-cured loins are considered. In practice, four product classes identified with different colored labels, namely: ‘white label’ (at least 50% Iberian breed, fed on concentrates), ‘green label’ (at least 50% Iberian breed, fed extensively on natural resources and concentrates), ‘red label’ (at least 50% Iberian breed, fed exclusively on natural resources) and ‘black label’ (100% pure Iberian breed, fed exclusively on natural resources).

The sensory analysis of foods provides solid and essential information related to the organoleptic profile of a product and/or information associated with consumer preference [4]. Among sensory methods, the descriptive analysis has been widely used, since it is possible to obtain a sensory profile by defining and quantifying the attributes related to the sample [5]. New and alternative sensory methods have emerged in sensory science to overcome the disadvantages and limitations of the more conventional or classic methods (as example time consumed, exhaustive panel training, or static responses), highlighting techniques such as Check-all-that-apply (CATA), Flash Profile (FP), Napping, or Temporal Dominance of Sensations (TDS) [6].

CATA is a relatively new technique that enables us to consider the ‘consumer voice’ from a sensory perspective [7] and with the advantage of being simple and easy to implement [8]. Moreover, the analysis of the CATA profile of the ‘Ideal product’ (the product that maximizes consumer liking) and the overall liking of studied samples improve the provided data obtained using this method [9]. Therefore CATA, considering liking and ‘Ideal’ products, presents important advantages in comparison with conventional techniques. This method is able to distinguish between the meat products regarding their organoleptic properties and indicate some important considerations in the elaboration of meat products [6,10]. CATA has been used with a range of meat products, such as bologna sausages [11], dry sausages [12], and mortadella [10]. The use of ‘CATA ideal’ can be helpful to identify the sensory characteristics of the ideal meat product according to the consumers’ perceptions, as indicated in a study by Saldaña et al. with bologna sausages [13], and by Peñaranda et al. [14] with chorizo.

It is also known that sensory perception is a dynamic phenomenon, as the nature and intensity of sensory attributes vary during the process of food consumption [15]. Dynamic sensory methods such as TDS are recently gaining importance since it allows the identification of the attribute perceived as ‘dominant’ throughout food consumption [16]. This technique has been used to evaluate meat products such bologna sausage [17], sausage [18], dry-cured loin [16], and dry-cured ham [19]. The original version of TDS only considers one intake, being a less-realistic approach of product consumption, while the multiple-intake/sip TDS technique allows series of concatenated TDS evaluations [20]. This version has recently been successfully applied to different products, providing more accurate and complete information in comparison with the original one [21]; however, there is still no work on using this approach with dry-cured loin.

Sensory characteristics and consumer perception of dry-cured loin and other related dry-cured products have been extensively studied [16,22]. However, a comprehensive study of the dominant attributes that characterize this product in a realistic consumption situation has not been carried out. Moreover, the identification of attributes acting as drivers of liking or disliking among consumers and the profiling of the ‘Ideal product’ could be very useful to gain additional insight into the relationship between the sensory profile and the consumer’s hedonic response to this meat product. Therefore, the aim of the present work was to identify the drivers of liking and disliking as well as the attributes that define the ideal dry-cured loin using CATA questionnaires. Moreover, the dominant sensory attributes during dry-cured loin consumption were identified by using the multiple intake TDS technique. Thus, this paper will bring the sensory characteristics of the Iberian dry-cured loin and its ideal version according to consumers’ perceptions, and a more realistic temporal profile with potential applicability in the food industry.

## 2. Materials and Methods

### 2.1. Samples

Three different commercial categories of dry-cured loins (total *n* = 9, 3 loins from each category) were purchased from Montesano Extremadura S.A. (Badajoz, Spain). The different categories (from cheaper to more expensive) were: ‘Cebo de Campo Ibérico with green label (GL), i.e., derived from 50% Iberian × Duroc crossbreed pigs extensively reared and fed on natural resources and concentrate, ‘Bellota 50% Ibérico’ with red label (RL), i.e., derived from 50% Iberian × Duroc crossbreed pigs extensively reared and exclusively fed on natural resources, and ‘Bellota 100%’ with black label (BL), i.e., derived from 100% Iberian pure breed pigs extensively reared and exclusively fed on natural resources. According to Spanish regulation [3], the dry-cured loin process consists of the following phases: pickling and stuffing in natural or artificial casings phase, and the dry-cured phase (minimum time 70 days). During the pickled phase, salt, spices (such as paprika) and condiments are added.

### 2.2. Participants

In study 1, Check-all-that-apply (CATA) analysis was carried out by one-hundred and twenty consumers, with ages ranging from 18 to 64 years old (52% were female and 48% were male), recruited at the University of Extremadura facilities. They included students (61%), and professors, administrative staff, researchers, and visitors (39%). In study 2, the multiple-intake TDS analysis was carried out by fifteen trained panelists (9 female and 6 male), with ages ranging from 21 to 59 years old and recruited from the Service of Innovation in Products of Animal Origin (SIPA) and the Meat and Meat Products Research Institute (IProCar) located at the Campus of the University of Extremadura (Cáceres). Participants had previous experience in conventional and dynamic sensory analysis of meat products, and all of them were previously trained in multiple TDS techniques [21]. Each of the fifteen panelists performed two replications of each product (i.e., 30 trials).

Prior to recruitment in both studies, participants were informed of the purpose of the research and the voluntary nature of participation, and an informed consent was obtained.

### 2.3. Study 1: Check All That Apply (CATA) Considering Ideal Product and Liking

Participants were asked to complete a CATA questionnaire with 19 terms related to the sensory characteristics of the dry-cured loins, selected based on previous works [22]. Selected attributes and definitions are shown in Table 1. Dry-cured loins were sliced using a slicer meat machine TGI 300 OMS S.r.l. (TGI, Jerago con Orago, Italy) (slice samples of 2 mm and 5 g) and were immediately served to the participants. Samples were individually presented on plates with a piece of unsalted cracker and a glass of mineral water to follow the rinsing protocol between samples. The presentation order of the samples was randomized. Attribute list order was also randomized across participants; therefore, each consumer received the CATA questionnaire with the terms in different order. This random structure is necessary, because there are attributes which could more easily catch participants attention [12]. Moreover, overall liking using a 5-point facial expression hedonic scale (1 = dislike very much, 5 = like very much) was presented to consumers for each sample. Finally, after sample evaluation, participants were asked to complete the same CATA questionnaire for their ‘Ideal’ Iberian dry-cured loin, with the following question: ‘Now, think about the ideal Iberian loin and mark those characteristics that it should have’. Only one session was necessary to collect the data from the 120 consumers, carrying out in small groups with a maximum 10 participants. The session was performed in the main hall of the Faculty of Veterinary at the University of Extremadura (Cáceres, Spain).

### 2.4. Study 2: Multiple-Intake TDS Technique

In order to select the attributes to be evaluated by multiple intake-TDS, three training sessions [5] were performed in which the three commercial categories of dry-cured loins were tasted. In these sessions, the total number of intakes, the total evaluation time, and the evaluated sensory attributes were fixed. Selected attributes and definitions are presented in Table 1. Following the study by Pineau et al. [23], eight attributes were selected and simultaneously presented to assessors. Sensory sessions were carried out in individual booths, under white light and with adequate ventilation. Samples were served on plates with three slices per batch (three intakes), along with a piece of unsalted cracker and a glass of mineral water to follow the rinsing protocol between samples. As aforementioned for the CATA procedure, the loins were sliced (2 mm thickness and 5 g) using a meat slicer machine TGI 300 OMS S.r.l. (TGI, Jerago con Orago, Italy) and immediately served to the panelists.

At the beginning of each TDS session, each panelist was asked to place the first portion of sample in the mouth and click the ‘start’ button to begin the evaluation. The panelists would then successively select on the computer screen the dominants attributes from the presented list. Attributes list and sample orders were randomized across the panel [24]. The selected TDS conditions for each dry cured loin were a total of 3 intakes and 100 s of total session time, with a 28 s interval for each intake, and with a ‘‘delay time’ of 2 s between each intake. After the third intake, the assessment stopped automatically at due time unless the panelist stopped it manually when dominant attributes were no longer perceived. FIZZ software 2.20 C version (Biosystèmes, Couternon, France, 2002) was used for collecting the data in multiple-intake TDS sessions.

### 2.5. Data Analysis

#### 2.5.1. CATA Considering Ideal Product and Liking

A contingency table was built with frequency of mention of each attribute for each group of samples (including the Ideal product). Cochran’s Q test was applied on the CATA results to study significant differences between samples (three commercial categories of Iberian dry-cured loins) for each of the attributes. Post hoc pairwise comparisons between samples were made using the critical difference (Sheskin) procedure, using the 5% level of significance (*p* < 0.05). Correspondence analysis (CA) was used to obtain the relationship between samples and terms from the CATA. In this analysis, the Ideal product was also considered. Moreover, a penalty analysis (PA) was performed on CATA data using the description of the Ideal and overall liking [25], considering the attributes mentioned by at least 20% of consumers. Finally, a Principal Coordinate Analysis (PCoA) was carried out on liking and CATA attributes.

Cochran’s Q test, Correspondence Analysis (CA), Penalty Analysis (PA) and Principal Coordinate Analysis (PCoA) from CATA evaluation were carried out using XLSTAT 2014 (Addinsoft, Paris, France). The overall liking data were also evaluated by ANOVA and Tukey’s Test at *p* < 0.05 using IBM SPSS software (v.22) (IBM Co., New York, NY, USA).

#### 2.5.2. Multiple-Intake TDS

TDS results were collected and analyzed using FIZZ software (v 2.20 C, Biosystèmes, Couternon, France). Line-based smoothing was applied to each curve. Additional lines, chance level and level of significance were included on each TDS curve. Chance level (P0) represents the dominance rate that an attribute can obtain by chance (P0 = 1/number of attributes), while the level of significance (Ps) represents the smallest value of the proportion being significantly (*p* = 0.05) higher than the chance level (Ps = P0 + 1.645[P0 (1 − P0)/n]1/2) where n is the number of runs: judges × replicates [23]. 

In addition, the TDS curves were assessed to compare the different categories of dry-cured loin, thus these differences were plotted only when they were significantly different from zero. Following the procedure described by Lenfant et al. [26], a Principal Component Analysis (PCA) using the texture and flavor % of Dominance Rate of each time-point and for each product intake was performed to show sample sensory trajectories over time. For this evaluation, 7 equally spaced time points were taken over the intake period of each sample, with a total of 5 points considered for each product intake. Sensory trajectories are shown by linking the time points corresponding to product evaluation: the first score (t_0) is the beginning of a sensory trajectory and the end point (t_88) corresponds to the last score before swallowing the sample. Moreover, the evaluation was divided into the three intakes. A mapping of the products was performed based on the first two principal components (PCA biplot) carried out using the XLSTAT 2014 (Addinsoft, Paris, France).

## 3. Results and Discussion

### 3.1. Sensory Characterization of Iberian Dry Cured Loins by CATA Analysis

**Figure 1:**
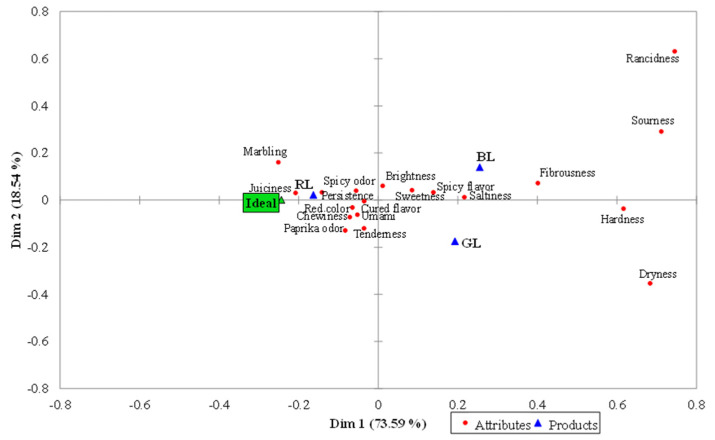
Correspondence Analysis (CA) of CATA term (attributes) frequencies for the three commercial categories of Iberian dry-cured loins: green label—GL (Cebo de Campo Ibérico), red label—RL (Bellota 50% Ibérico), black label—BL (Bellota 100%) and Ideal.

Table 2 displays the frequency of mention (%) of each term using the CATA method, and the CA plot is shown in Figure 1. Fourteen of nineteen attributes of the CATA questionnaire showed significant differences among the three categories of dry-cured loin evaluated and the Ideal (Table 2). Participants cited with a significantly higher frequency the terms juiciness, chewiness, marbling, and redness color in RL samples and the Ideal product compared to BL and GL dry-cured loins. Moreover, BL dry cured loins were characterized by a significant highest frequency of mention for rancidity. These rancid notes are not negatively associated with sensory characteristics of dry-cured products, as long as they are not dominant and therefore are balanced by other pleasant flavor notes [27]. RL and the Ideal showed lower citation frequencies for hardness and fibrousness although these differences were not significant compared to GL samples. In concordance to juiciness results, frequency of mention of dryness was significantly lower in RL and Ideal compared to GL and BL. Finally, GL samples did not show significant differences for sourness, hardness, saltiness, fibrousness, paprika flavor, chewiness, and tenderness compared to RL and BL dry-cured loins. CA plot (Figure 1) shows the differences in the sensory map among the evaluated samples and the Ideal. Two first dimensions explained the 92.13% (75.59% for the dimension 1, and 18.54% for the dimension 2) of the total variance. Each group of samples was located in different quadrants, showing a different sensory map. RL was mainly related to juiciness, persistence, spicy odor, umami, cured flavor, red color and marbling while this sample is located in the opposite quadrant to dryness. GL was located close to tenderness, umami, chewiness and saltiness and in the opposite place to marbling attribute. BL samples were located in the same quadrant than saltiness, fibrousness, spicy flavor, sweetness, brightness, sourness and fibrousness. Regarding the Ideal, it is located near to RL dry cured loin samples, showing a similar sensory profile. According to these results, consumers highly valued attributes such as juiciness, persistence or marbling, explaining the similar location of RL and the Ideal. Despite being, ‘a priori’, the most valuable commercial category, BL was situated far from the Ideal and associated with other attributes such as spicy flavor, fibrousness or saltiness, for example.

**Figure 2:**
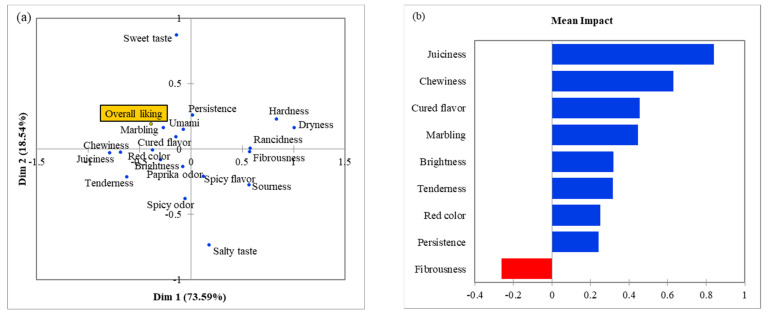
Principal Coordinate Analysis (PCoA) plot (**a**) of CATA term frequencies and overall liking scores for the dry-cured loin samples: green label—GL (Cebo de Campo Ibérico), red label—RL (Bellota 50% Ibérico), black label—BL (Bellota 100%), and liking mean impact plot (**b**) displaying in blue attributes identified as ‘drivers of liking’ and in red the ‘drivers of disliking’ attributes.

RL samples obtained the highest overall liking values (4.07 ± 0.06) in comparison with GL (3.54 ± 0.07), and BL (3.58 ± 0.09). The representation of the sensory attributes that drive the overall linking is displayed on Figure 2a. Overall liking scores seemed to be positively correlated with attributes that were linked to the Ideal product in correspondence analysis, therefore several attributes of RL were also correlated. Cured flavor, umami and marbling were closely located to overall linking and were highly influential in consumer acceptability. Cured flavor was also highlighted in other studies about Iberian dry-cured loins [2]. On the same line, marbling is also considered a quality index by other participants [1].

Figure 2b displays the liking mean impact plot in which attributes with significant impact on liking are showed. Therefore, juiciness, chewiness, cured flavor, marbling, brightness, tenderness, red color, and persistence were drivers of liking. Meanwhile, fibrousness negatively influenced the mean impact, noted as a driver of disliking. It is remarkable that these attributes were also found in the CA plot, associated with Ideal loin as well as RL samples. These drivers of liking have already been highlighted as relevant attributes in other sensory studies of dry cured loin [2,16].

### 3.2. Penalty Analysis Based on CATA Questionnaire Considering Ideal Product

**Figure 3:**
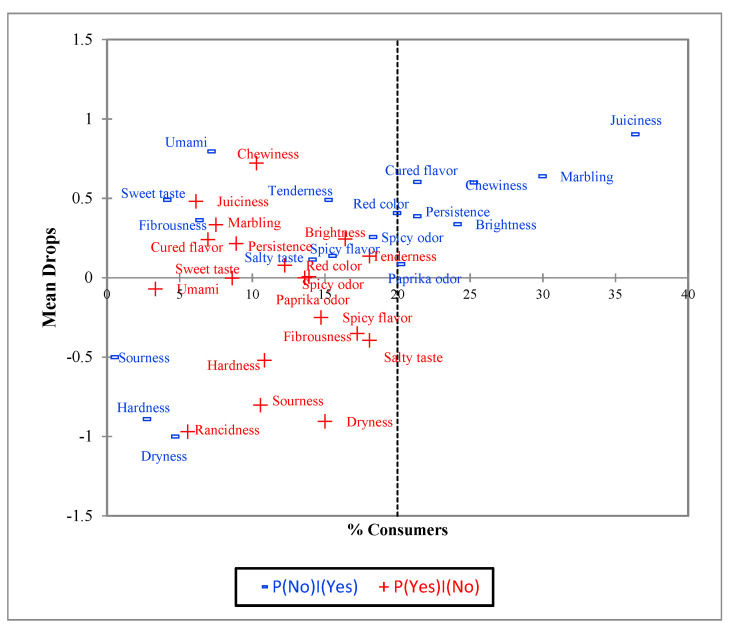
Penalty Analysis (PA) from CATA with ideal data, in which the attribute is selected for the ideal product, but missing in the real products (‘must have attributes) (blue terms), and in which the attribute is not selected for the ideal, but endorsed for the real products (‘nice to have’, ‘must not have’, ‘does not harm’ attributes) (red terms).

For PA analysis, the instructions described by Meyners et al. [28] were followed, considering the data of CATA of the three studied samples and the Ideal product. These instructions are based on the proportion of selection between real and Ideal products and how this proportion impacts the liking (congruence, incongruence). Thus, the attribute could be ‘must have’, ‘nice to have’, ‘do not harm’, or ‘must not have’. Figure 3 shows the graphical representation of PA analysis from CATA with Ideal product data. Only ‘must have’ and ‘do not harm’ were obtained. Juiciness, marbling, cured flavor, chewiness, persistence, and brightness were the ‘must have’ terms for the consumers. These attributes defined the Ideal loin, and therefore had a significant mean impact. The flavor results were consistent with the literature, since curing and after-taste flavor (persistence) have been previously highlighted by other authors as relevant flavor attributes in dry-cured loins [1]. The other attributes did not show significant influence on the mean impact and were classified as ‘do not harm’ attributes. 

According to the aforementioned data, RL was, among dry-cured loins, the closest to the Ideal loin, characterized by a noticeable cured flavor and persistence. Additionally, it is remarkable that consumers preferred dry-cured loins less salted and fibrous (Table 2).

### 3.3. Dynamic Sensory Characterization of Iberian Dry-Cured Loins: Multiple Intake TDS Approach

**Figure 4:**
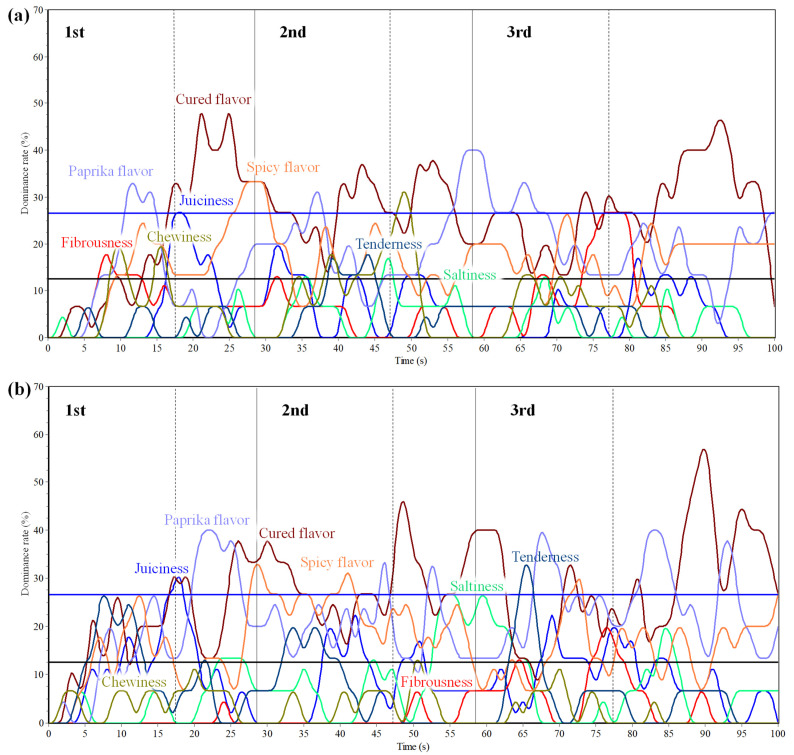

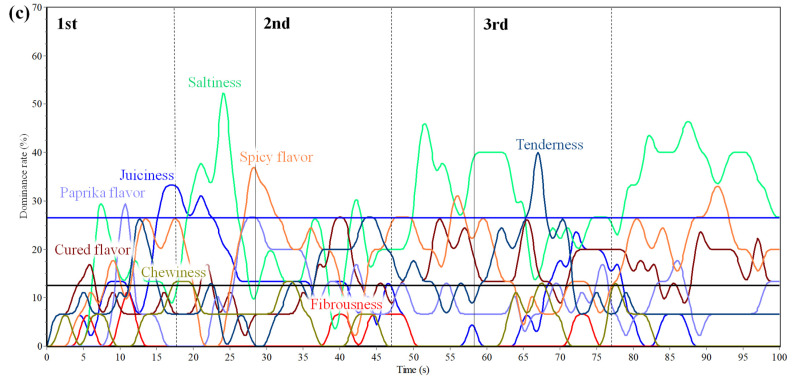
Multiple-intake TDS dominance curves of eight sensory attributes during three intakes of GL (**a**); RL (**b**); BL (**c**) dry cured loins (GL = green label, Cebo de Campo Ibérico dry-cured loin; RL = red label, Bellota 50% Ibérico dry-cured loin; BL= black label, Bellota 100% dry-cured loin). Two horizontal lines account for chance level (▬) and significance level (▬) and two vertical lines indicate the time of each intake (─) and the moment of swallowing (---).

Figure 4a–c displays multiple-intake TDS curves for the three evaluated commercial categories of dry-cured loin. Each curve represents the evolution of the dominance rate of an attribute over time. Chance (12.5%) and significance (26.5%) levels are shown on the curves.

Figure 4a shows the TDS curves obtained over three consecutive intakes of GL dry-cured loin. Paprika, cured, and spicy flavors significantly dominated the first intake; paprika flavor, chewiness and cured flavor dominated the second intake whereas paprika flavor, cured flavor and fibrousness were the dominant attributes along the third intake. Paprika flavor was chosen as the first significant dominant sensation by 32% of the panel at 11 s of product evaluation, and was also the most dominant attribute along the second intake, with a maximum of 40% of dominance rate at 58 s. Cured flavor was the longest significant dominant attribute along the three intakes, showing its maximum at 20 s with a 47% of dominance rate. A similar evolution was observed along the three intakes since paprika flavor was always the first dominant attribute followed by a cured flavor.

Figure 4b displays the multiple-intake TDS curves of the RL sample. The first and third intake were significantly dominated by four attributes, while the second one by three. Cured, paprika and spicy flavor were presented along the three intakes; juiciness also characterized the first intake, and tenderness the third one. Cured flavor was selected as the first significant dominant sensation, with a dominant rate of 30% at 16 s of the first intake. Paprika flavor was the most dominant attribute for the first intake displaying a maximum of 40% dominant rate. Moreover, cured flavor dominated the third intake. This attribute showed the maximum dominance rate at 88 s on the third intake, with 55%. As reported in the literature [29], dry-cured loins from pigs fed only on acorns show pleasant and intense flavor attributes, which is consistent with the current results. 

Finally, Figure 4c shows the BL multiple intake TDS curves. The first intake was characterized by four attributes, namely, saltiness, paprika flavor, juiciness, and spicy flavor. The second intake was dominated by spicy flavor, tenderness, cured flavor and saltiness while the third intake was dominated by saltiness, tenderness, and spicy flavor. Saltiness was selected as the first significant dominant sensation perceived by the panel, with a 29% of dominance rate at 6 s. Moreover, saltiness was also the most dominant attribute in each intake, with a maximum of 53% of dominance rate at 25 s (first intake Paprika and spicy flavor attributes were dominant at the beginning of the intakes, subsequently followed by cured flavor and saltiness, which dominated and probably masked the perception of other sensory attributes. Similarly, other authors such as Lorido et al. [16], reported that cured flavor and saltiness had a great masking effect on dry-cured loin temporal profiles.

**Figure 5:**
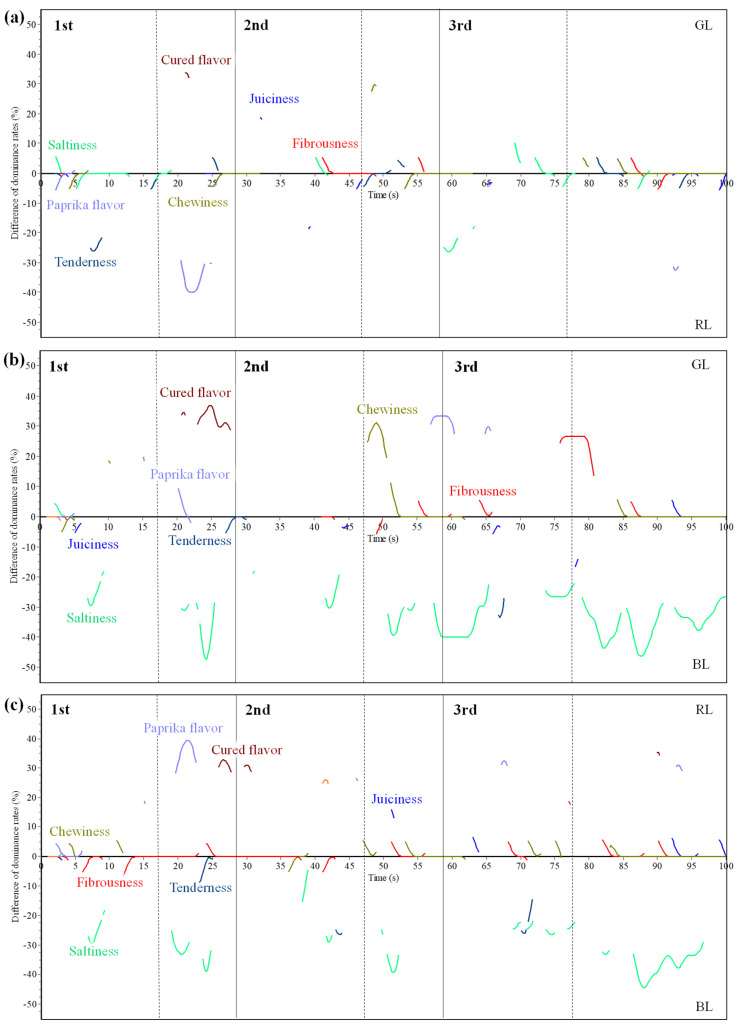
Difference Multiple-intake TDS curves of GL vs. RL (**a**), GL vs. BL (**b**), and RL vs. BL (**c**), filtered with a level of significance (5%) (GL = green label, Cebo de Campo Ibérico dry-cured loin; RL = red label, Bellota 50% Ibérico dry-cured loin; BL= black label, Bellota 100% dry-cured loin).

Figure 5 shows the difference TDS curves of dominant sensations between GL vs. RL (a), GL vs. BL (b), and RL vs. BL (c) per intake. Considering Figure 5a, saltiness, chewiness, tenderness, juiciness, and fibrousness attributes were found to be dominant in both loins at particular intakes. However, paprika flavor was only dominant in RL and cured flavor in GL dry-cured loins during the first intake. It is also noted that juiciness and saltiness showed significant differences between sample along the third intake. Concerning GL vs. BL TDS differences curves (Figure 5b), saltiness, as expected, displayed significant differences among batches, since BL temporal profile was highly characterized by the significant dominance of this attribute in all intakes. On the other hand, GL was significantly dominated by cured flavor during the first intake, by chewiness and paprika flavor in the second one, and finally by fibrousness in the third intake. Finally, Figure 5c displays the differences TDS curves of RL vs. BL. Once again, saltiness was significantly highlighted by BL, dominating in all intakes. Saltiness has been reported by other authors to mask other sensory attributes in dry-cured loins [16]. Moreover, tenderness showed higher dominance in BL compared to RL, which could be due to the higher intramuscular fat content usually reported for dry-cured meat products derived from Iberian pure breed pigs [1]. The temporal profile of RL was characterized by paprika and cured flavor, juiciness and fibrousness. 

**Figure 6:**
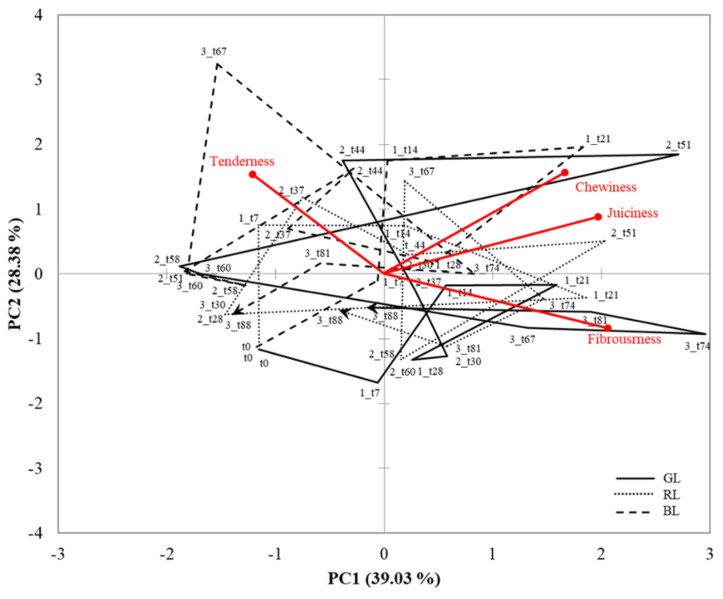
Principal Component Analysis representing the texture trajectories of the dry-cured loin samples (GL = green label, Cebo de Campo Ibérico dry-cured loin; RL = red label, Bellota 50% Ibérico dry-cured loin; BL= black label, Bellota 100% dry-cured loin) over the three intakes.

The sensory trajectories related to texture attributes perception are represented in Figure 6. First two PCA components account for 67.41% of the total variance. The chewiness and juiciness attribute vectors were very close in the representation, indicating a positive correlation, while fibrousness and tenderness were located in opposite quadrants showing negative correlations with PC2 and PC1, respectively. The trajectories did not converge towards one point in any of the three intakes for the three samples. Despite this fact, differences between samples were found. GL loins were characterized by fibrousness at some points of the evaluation. The dominance of this attribute could explain the low overall liking values of GL since fibrousness was considered a driver of dislike. GL and BL loins showed dominance of chewiness and juiciness, in the second and first intake, respectively, while RL converged to these attributes in several points of the evaluation, but with lower dominance rate values. Regarding tenderness, BL converged to this attribute in the third intake, whereas RL and GL were characterized by a dominance of tenderness along the first two intakes. It is also remarkable that sensory trajectory of BL samples did not converge to fibrousness at any moment of the evaluation.

**Figure 7:**
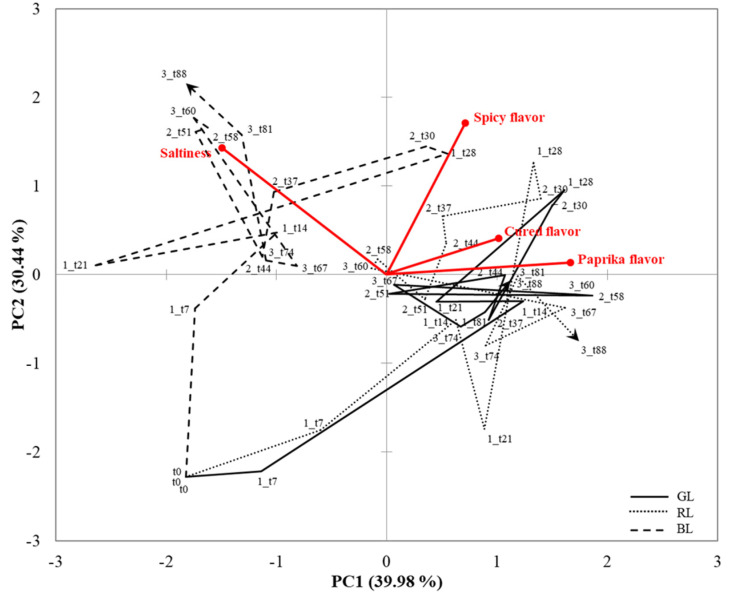
Principal Component Analysis representing the flavor trajectories of the dry-cured loin samples (GL = green label, Cebo de Campo Ibérico dry-cured loin; RL = red label, Bellota 50% Ibérico dry-cured loin; BL= black label, Bellota 100% dry-cured loin) over the three intakes.

The sensory trajectories related to flavor attribute perception are represented in Figure 7. The first two PCA components account for 70.42% of the total variance. Spicy, cured, and paprika flavor vectors were located in the upper-right quadrant, meanwhile saltiness was situated in the upper-left quadrant. The distribution of the trajectories showed differences among BL and, GL and RL sample locations. BL was clearly different and was characterized by a dominance of saltiness during the three intakes, particularly along the second and third intake and converged to this attribute at the end. On the other hand, GL and RL did not show any influence of saltiness. Sensory trajectories of these sample converged to cured and paprika flavor particularly along the second and the third intake. In addition, and particularly during the end of the first intake and the second one, sensory trajectories of BL dry-cured loins converged to spicy flavor. Trajectory plots supported the results obtained by TDS curves, remarking again the main differences between samples, as for example the aforementioned greater saltiness dominance for BL. 

### 3.4. Comparison of CATA and TDS Results

As previously reported, the Ideal Iberian dry-cured loin must be juicy, chewy, with a persistent flavor, bright, with visible presence of intramuscular fat and cured flavor. These attributes were also highly cited in the CATA questionnaires of RL dry-cured loin and we can consider that the sensory profile of these samples was the closest to the Ideal dry-cured loin. Results from dominant attributes of TDS curves for RL also support the importance of these attributes particularly those related to flavor and agreeing with previous works [16]. Moreover, dominance of cured flavor increased along the intakes, showing that the multiple-intake TDS approach offered a more accurate information related to the temporal sensory changes, compared to classical TDS with only one intake [30]. Regarding the GL batch, once again flavor attributes were highlighted by TDS curves. Cured, paprika, and spicy flavor were dominant attributes, without presenting any trends along TDS curves. These attributes presented the higher dominance rate values and thus were close to the Ideal one. CATA analysis allowed us to distinguish between dry-cured loin categories in terms of texture attributes whereas temporal TDS profile of juiciness and chewiness were not significantly different among samples. Finally, BL were clearly dominated and characterized by the saltiness attribute. Cochran’s Q test (CATA data) and TDS results agree that this attribute was one of the most noticeable attributes for BL samples and probably affected the perception of the rest of the attributes. Therefore, this could be the reason why BL was not located close to the Ideal product. Some texture parameters were dominant during TDS evaluation, such as juiciness and tenderness, probably associated with fat content [2]. 

As was reported by Agudelo et al. [8], for a better understanding of the sensory perception it has been necessary for the application of the different sensory techniques, which thus complement the information obtained by each method. For the characterization of the different categories of dry-cured loins, CATA and multiple-intake TDS were well complemented and supported between them. Therefore, the combination of these sensory methods has been demonstrated to be efficient for a sensory characterization and discrimination of different commercial categories of dry-cured loins.

## 4. Conclusions

CATA analysis considering Ideal product and overall liking, and multiple-intake TDS, allowed further examination of consumer perception. Both techniques provided solid sensory data of commercial Iberian dry-cured loins with different genetic and rearing backgrounds, identifying the most remarkable attributes as well as describing the temporal profile in a more realistic consumption situation. RL, despite not being 100% Iberian, was selected as the closest to the Ideal Iberian dry-cured loin for consumers. Attributes such as juiciness, chewiness, or cured flavor, had a positive influence on consumers’ liking, while fibrousness showed the opposite effect. Several of these drivers of liking were considered must-have attributes and significantly dominated RL TDS curve.

This study shows for a first time the feasibility of applying rapid and temporal sensory techniques to effectively sensorily characterize different commercial categories of Iberian dry-cured loins, being complementary and consistent with each other. Both sensory methodologies provided a different sensory approach which could be useful in different stages of product development and for marketing purposes.

## Figures and Tables

**Table 1:** Attributes and descriptions used on CATA and multiple-intake TDS sessions.

Technique	Attribute	Description
CATA	Red color ^a^	Redness of the sample
	Brightness ^a^	Reflection of light
	Marbling ^a^	Visible intramuscular fat
	Paprika odor ^o^	Odor perception characteristic of paprika
	Spicy odor ^o^	Odor perception characteristic of pepper, garlic and nutmeg
	Hardness ^te^	Big effort required to convert the sample in a swallowable state
	Tenderness ^te^	Ease effort required to convert the sample in a swallowable state
	Chewiness ^te^	Number of chews until to reach a state ready for swallowing
	Fibrousness ^te^	Fiber perception during chewing
	Juiciness ^te^	Impression of lubricated food during chewing
	Dryness ^te^	Free from moisture perception
	Persistence ^te^	Continued flavor perception after swallowing the sample
	Saltiness ^ta^	Primary salty taste
	Umami taste ^ta^	Primary umami taste
	Sweetness ^ta^	Primary sweet taste
	Sourness ^ta^	Primary acid taste
	Rancidity ^f^	Flavor perception characteristic of oxidized pork fat
	Spicy flavor ^f^	Spicy flavor characteristic of pepper, garlic and nutmeg
	Cured flavor ^f^	Flavor perception characteristic of cured meat products
Multiple-intake TDS	Juiciness ^te^	Impression of lubricated food during chewing
	Fibrousness ^te^	Fiber perception during chewing
	Chewiness ^te^	Number of chews until to reach a state ready for swallowing
	Tenderness ^te^	Ease effort required to convert the sample in a swallowable state
	Saltiness ^ta^	Primary salty taste
	Cured flavor ^f^	Flavor perception characteristic of cured meat products
	Paprika flavor ^f^	Flavor perception characteristic of paprika
	Spicy flavor ^f^	Spicy flavor characteristic of pepper, garlic and nutmeg

^a^ = appearance; ^o^ = odor; ^te^ = texture; ^f^ = flavor; ^ta^ = taste; CATA: Check-all-that-apply; TDS: Temporal Dominance of Sensations.

**Table 2:** Frequency of mention (%) of each attribute by consumers using CATA questionnaires for the three commercial categories of dry-cured loins and ideal product.

Attributes	Samples			
	GL	RL	BL	IDEAL
Red color ***	53 ^a^	67 ^b^	50 ^a^	63 ^b^
Brightness ^ns^	44	52	57	58
Marbling ***	20 ^a^	51 ^c^	38 ^b^	58 ^c^
Paprika odor *	35 ^ab^	37 ^b^	24 ^a^	38 ^b^
Spicy odor ^ns^	23	32	28	32
Hardness **	14 ^ab^	5 ^a^	16 ^b^	3 ^a^
Tenderness ***	46 ^ab^	53 ^b^	33 ^a^	40 ^ab^
Chewiness **	66 ^ab^	73 ^b^	55 ^a^	78 ^b^
Fibrousness *	20 ^ab^	14 ^a^	27 ^b^	9 ^a^
Juiciness ***	46 ^a^	76 ^b^	51 ^a^	87 ^b^
Dryness ***	26 ^b^	3 ^a^	18 ^b^	5 ^a^
Persistence **	21 ^a^	28 ^ab^	24 ^ab^	37 ^b^
Saltiness **	39 ^ab^	28 ^a^	46 ^b^	33 ^a^
Umami ^ns^	13	14	12	16
Sweetness ^ns^	11	16	13	8
Sourness **	9 ^ab^	6 ^a^	18 ^b^	1 ^a^
Rancidity **	3 ^a^	3 ^a^	11 ^b^	0 ^a^
Spicy flavor ^ns^	30	24	37	31
Cured flavor **	38 ^a^	38 ^a^	40 ^ab^	52 ^b^

Attributes which frequencies differ between samples at *** *p* ≤ 0.001; ** *p* ≤ 0.01; * *p* ≤ 0.05; ^ns^ no significant (*p* > 0.05). Values within rows with different lowercase superscripts are significantly different according to critical difference (Sheskin) paired comparisons test (at *p* < 0.05). GL = green label, Cebo de Campo Ibérico dry-cured loin; RL = red label, Bellota 50% Ibérico dry-cured loin; BL= black label, Bellota 100% dry-cured loin.

## Data Availability

The datasets generated for this study are available on request to the corresponding author.
